# Crystal structure of 1,4,8,11-tetra­azonia­cyclo­tetra­decane bis­(dichromate) monohydrate from synchrotron data

**DOI:** 10.1107/S2056989017005771

**Published:** 2017-04-28

**Authors:** Dohyun Moon, Jong-Ha Choi

**Affiliations:** a Pohang Accelerator Laboratory, POSTECH, Pohang 37673, Republic of Korea; b Department of Chemistry, Andong National University, Andong 36729, Republic of Korea

**Keywords:** crystal structure, 1,4,8,11-tetra­azonia­cyclo­tetra­deca­ne, dichromate anion, hydrogen bonding, synchrotron radiation

## Abstract

The asymmetric unit of the hydrated title salt, (C_10_H_28_N_4_)[Cr_2_O_7_]_2_·H_2_O [C_10_H_28_N_4_ = H_4_(cyclam) = 1,4,8,11-tetra­azonia­cyclo­tetra­deca­ne], contains two half-cations (both completed by crystallographic inversion symmetry), two dichromate anions and one water mol­ecule. The two [CrO_7_]^2−^ anions exhibit a nearly staggered conformation, with bridging angles of 133.37 (11) and 136.28 (12)°. The distortions of the dichromate anions are due to their participation in hydrogen-bonding inter­actions with the water mol­ecule and the cations. Inter­molecular hydrogen bonds involving the cyclam N—H groups and water O—H groups as donor groups, and the O atoms of the dichromate anions as acceptor groups give rise to a three-dimensional network.

## Abstract 2

In the title hydrated salt, (C_10_H_28_N_4_)^4+^·2[Cr_2_O_7_]^2−^·H_2_O, the two unique cations lie about an inversion centre. In the crystal, O—H⋯O and N—H⋯O hydrogen bonds connect the anions, cations and water mol­ecule, forming a three-dimensional network

## Chemical context   

Chromium(VI) compounds are highly cytotoxic and potential carcinogens (Cohen *et al.*, 1993 ▸). A number of treatment methods for the removal of such toxic heavy metal ions in water have been described (Kalidhasan *et al.*, 2016 ▸), and 1,4,8,11-tetra­aza­cyclo­tetra­decane (cyclam) is possibly one of the most useful candidates for this purpose since it has a strong ability to act as an effective metal-ion binding mol­ecule. The aza­macrocycle is a strong basic amine to form a dication, (C_10_H_26_N_4_)^2+^, or a tetra­cation, (C_10_H_28_N_4_)^4+^, in both of which all of the N—H bonds are generally active in hydrogen-bond formation. These di- or tetra­ammonium cations may also be suitable candidates for the removal of toxic metal ions. Previously, the syntheses and crystal structures of [H_2_(cyclam)](ClO_4_)_2_ (Nave & Truter, 1974 ▸), [H_2_(cyclam)]Cl_2_·0.5H_2_O (Kim *et al.*, 2009 ▸), [H_4_(cyclam)](NO_3_)_4_·2H_2_O (Harrowfield *et al.*, 1996 ▸), [H_2_(cyclam)](maleate)_2_ (Mireille Ninon *et al.*, 2013 ▸), [H_4_(cyclam)](HSO_4_)_4_ (Said *et al.*, 2013 ▸), [H_4_(cyclam)]Cl_4_, [H_4_(cyclam)]Cl_4_·4H_2_O, [H_4_(cyclam)]Br_4_·4H_2_O, [H_4_(cyclam)](ClO_4_)_4_·2H_2_O (Robinson *et al.*, 1989 ▸) and [H_4_(cyclam)](SO_4_)_2_·6H_2_O (Subramanian & Zaworotko, 1995 ▸) have been reported. The crystal structure of neutral cyclam has also been determined (Robinson *et al.*, 1989 ▸), but a combination of the 1,4,8,11-tetra­azonia­cyclo­tetra­decane cation with the [CrO_7_]^2−^ anion has not been reported. We give here details of the preparation of the title compound, a new hydrated organic dichromate(VI) salt, [H_4_(cyclam)][Cr_2_O_7_]_2_·H_2_O, (I), and its structural characterization by synchrotron single-crystal X-ray diffraction.
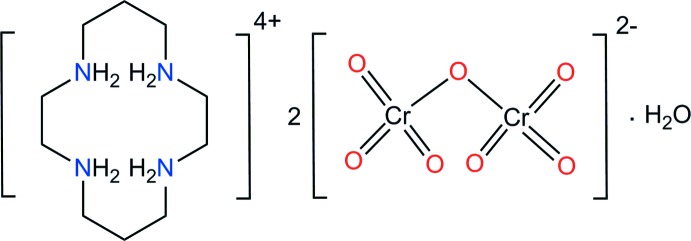


## Structural commentary   

**Figure 1:**
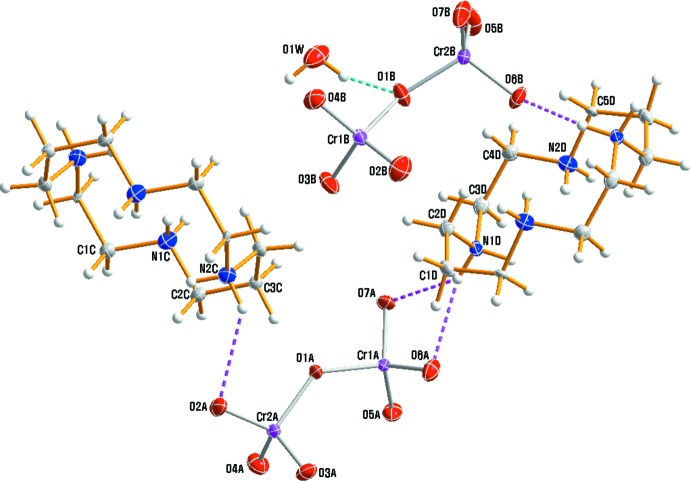
The structures of the mol­ecular components in (I), drawn with displacement ellipsoids at the 60% probability level. Dashed lines represent hydrogen-bonding inter­actions.

An ellipsoid plot of the mol­ecular components of (I) along with the atom-numbering scheme is shown in Fig. 1 ▸. The asymmetric unit comprises of two half-cations (both completed by crystallographic inversion symmetry), two dichromate anions and one water mol­ecule. Within the centrosymmetric tetra-protonated amine unit, (C_10_H_28_N_4_)^4+^, the C—C and N—C bond lengths range from 1.491 (3) to 1.520 (3) Å and from 1.489 (3) to 1.524 (3) Å, respectively. The range of N—C—C and C—N—C angles is 109.84 (19) to 116.69 (18)° and 110.15 (18) to 111.5 (2)°, respectively. Bond lengths and angles within the tetra­ammonium cations are comparable to the corresponding values determined for the cyclam ligand in *trans*-[Cr(nic-O)_2_(cyclam)]ClO_4_ (nic-O = O-coordinating nicotinate; Choi, 2009 ▸), *cis*-[Cr(ONO)_2_(cyclam)]NO_2_ (Choi *et al.*, 2004*a* ▸), [Cr(ox)(cyclam)]ClO_4_ (ox = oxalate; Choi *et al.*, 2004*b* ▸), [Cr(acac)(cyclam)](ClO_4_)_2_·0.5H_2_O (acac = acetyl­acetonate; Subhan *et al.*, 2011 ▸), *cis*-[Cr(NCS)_2_(cyclam)]NCS (Moon *et al.*, 2013 ▸) or [CrCl_2_(cyclam)][Cr(ox)(cyclam)](ClO_4_)_2_ (Moon & Choi, 2016 ▸).

It is of inter­est to compare the conformation of the [CrO_7_]^2−^ anion with those found in other ionic crystals. In (I), the two [CrO_7_]^2−^ anions exhibit a nearly staggered conformation whereas an eclipsed conformation is observed for (C_3_H_5_N_2_)(NH_4_)[Cr_2_O_7_] or (C_9_H_14_N)_2_[Cr_2_O_7_] (Zhu, 2012 ▸; Trabelsi *et al.*, 2015 ▸). The conformation of dichromate anions appears to show a dependence on the size of the associated counter-cation (Moon *et al.*, 2015 ▸, 2017 ▸). The Cr1*A*—O1*A*—Cr2*A* and Cr1*B*—O1*B*—Cr2*B* bridging angles in the anions in (I) are 133.37 (11) and 136.28 (12)°, respectively, slightly larger than 130.26 (10)° in [Cr(urea)_6_][Cr_2_O_7_]Br·H_2_O (Moon *et al.*, 2015 ▸). The smaller Cr1*A*—O1*A*—Cr2*A* bridging angle is probably due to the non-involvement of the terminal oxygen atoms of Cr2*A* in any hydrogen bond. Cr—O_b_ (O_b_ = bridging O atom) bonds range from 1.7711 (19) to 1.799 (2) Å while the Cr—O_t_ bond lengths to the terminal O atoms vary from 1.590 (2) to 1.6417 (19) Å, with a mean terminal Cr—O bond length of 1.615 Å. The Cr—O bond lengths for atoms involved in hydrogen-bonding inter­actions are slightly longer than the other Cr—O bonds. This trend is similar to that observed for comparable anions in the structures of [Cr(urea)_6_][Cr_2_O_7_]Br·H_2_O (Moon *et al.*, 2015 ▸), [Cr(NCS)_2_(cyclam)]_2_[Cr_2_O_7_]·H_2_O (Moon *et al.*, 2017 ▸) or [Cr(ox)(cyclam)]_2_[Cr_2_O_7_]·8H_2_O (Moon & Choi, 2017 ▸).

## Supra­molecular features   

**Figure 2:**
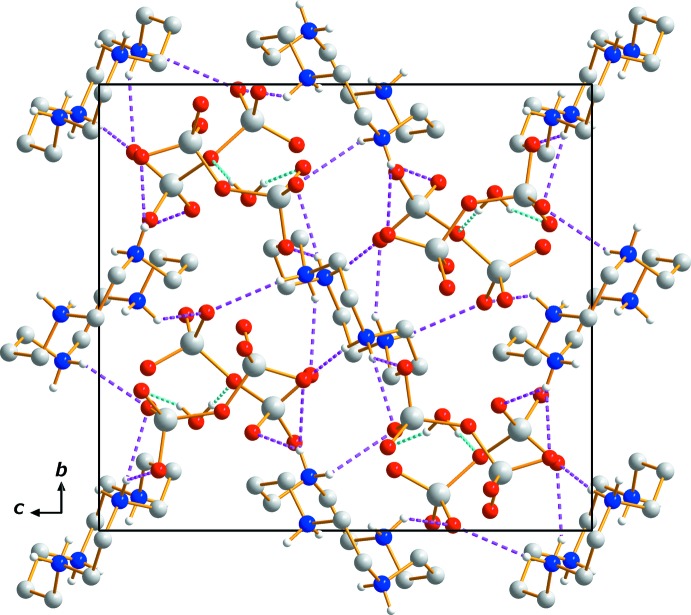
The crystal packing in compound (I), viewed perpendicular to the *bc* plane. Dashed lines represent N—H⋯O (pink) and O—H⋯O (cyan) hydrogen-bonding inter­actions, respectively. H atoms bound to C atoms have been omitted.

Extensive N—H⋯O and O—H⋯O hydrogen-bonding inter­actions occur in the crystal structure (Table 1 ▸). Two O—H⋯O hydrogen bonds link the water mol­ecule to two neighboring [CrO_7_]^2−^ anions while N—H⋯O hydrogen bonds inter­connect the (C_10_H_28_N_4_)^4+^ cations with both anions (Figs. 1 ▸ and 2 ▸). An extensive array of these contacts generates a three-dimensional network (Fig. 2 ▸) and, apart from Coulombic inter­actions, these hydrogen-bonding inter­actions help to stabilize the crystal structure.

## Database survey   

A search of the Cambridge Structural Database (Version 5.38, Feb 2017 with two updates; Groom *et al.* 2016 ▸) revealed a total of 24 hits for compounds containing 1,4,8,11-tetra­azonia­cyclo­tetra­decane (C_10_H_28_N_4_)^4+^ or 4,11-di­aza-1,8-diazo­nia­cyclo­tetra­decane (C_10_H_26_N_4_)^2+^ cations, but a combination with dichromate anions has not been reported.

## Synthesis and crystallization   

Cyclam (98%) was purchased from Sigma–Aldrich and used without further purification. All other chemicals were reagent-grade materials, and were used as received. 0.102 g of chromium trioxide (1 mmol, Sigma–Aldrich) was dissolved in 20 ml of water and 0.012 g of cyclam (0.06 mmol, Sigma–Aldrich) was added at room temperature. The mixture was stirred for 30 minutes and the resulting solution was filtered. The neat filtrate was allowed to stand for one week to give block-like yellow crystals of (I) suitable for X-ray structural analysis.

## Refinement   

Crystal data, data collection and structure refinement details are summarized in Table 2 ▸. All hydrogen atoms were placed in geometrically idealized positions and constrained to ride on their parent atoms, with C—H = 0.99 Å and N—H = 0.91 Å, respectively, and with *U*_iso_(H) values of 1.2*U*_eq_ of the parent atoms. The hydrogen atoms of the solvent water mol­ecule were assigned based on a difference-Fourier map, and were refined with distance restraints of 0.84 (2) Å (using DFIX and DANG commands), and with *U*_iso_(H) values of 1.5*U*_eq_ of the parent atom. The remaining maximum and minimum electron densities in the final Fourier map are located 0.85 and 0.54 Å, respectively, from the Cr1*B* site. Six reflections with a poor agreement between measured and calculated intensities were omitted from the final refinement cycles.

## Supplementary Material

Crystal structure: contains datablock(s) I. DOI: 10.1107/S2056989017005771/wm5385sup1.cif

Structure factors: contains datablock(s) I. DOI: 10.1107/S2056989017005771/wm5385Isup2.hkl

Supporting information file. DOI: 10.1107/S2056989017005771/wm5385Isup3.cml

CCDC reference: 1534082

Additional supporting information:  crystallographic information; 3D view; checkCIF report

## Figures and Tables

**Table 1:** Hydrogen-bond geometry (Å, °)

*D*—H⋯*A*	*D*—H	H⋯*A*	*D*⋯*A*	*D*—H⋯*A*
N1*C*—H1*NC*⋯O2*B* ^i^	0.91	2.45	3.091 (3)	128
N1*D*—H1*ND*⋯O6*A*	0.91	2.38	3.140 (3)	142
N1*D*—H1*ND*⋯O7*A*	0.91	2.16	2.942 (3)	143
N1*D*—H2*ND*⋯O6*B* ^ii^	0.91	1.87	2.768 (3)	169
N2*C*—H3*NC*⋯O6*A* ^iii^	0.91	2.62	3.074 (3)	112
N2*C*—H4*NC*⋯O7*B* ^iv^	0.91	2.42	3.047 (3)	126
N2*C*—H4*NC*⋯O2*A*	0.91	2.52	3.200 (3)	132
N2*D*—H3*ND*⋯O6*B* ^ii^	0.91	2.42	3.198 (3)	144
N2*D*—H4*ND*⋯O2*B* ^ii^	0.91	2.65	3.357 (3)	136
O1*W*—H1*O*1⋯O5*A* ^v^	0.84 (1)	2.38 (10)	2.999 (3)	130 (11)
O1*W*—H2*O*1⋯O1*B*	0.84 (1)	2.05 (4)	2.774 (3)	143 (5)

Symmetry codes: (i) 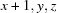; (ii) 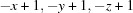; (iii) 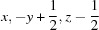; (iv) 
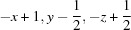
; (v) 
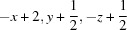
.

**Table 2:** Experimental details

Crystal data	
Chemical formula	(C_10_H_28_N_4_)[Cr_2_O_7_]_2_·H_2_O
*M*_r_	654.38
Crystal system, space group	Monoclinic, *P*2_1_/*c*
Temperature (K)	200
*a*, *b*, *c* (Å)	10.428 (2), 13.961 (2), 15.490 (2)
β (°)	94.671 (3)
*V* (Å^3^)	2247.6 (6)
*Z*	4
Radiation type	Synchrotron, λ = 0.610 Å
μ (mm^−1^)	1.28
Crystal size (mm)	0.11 × 0.10 × 0.09

Data collection	
Diffractometer	ADSC Q210 CCD area detector
Absorption correction	Empirical (using intensity measurements) (*HKL3000sm *SCALEPACK**; Otwinowski & Minor, 1997 ▸)
*T*_min_, *T*_max_	0.942, 1.000
No. of measured, independent and observed [*I* > 2σ(*I*)] reflections	12953, 6614, 5804
*R*_int_	0.016
(sin θ/λ)_max_ (Å^−1^)	0.706

Refinement	
*R*[*F* ^2^ > 2σ(*F* ^2^)], *wR*(*F* ^2^), *S*	0.038, 0.124, 1.09
No. of reflections	6614
No. of parameters	307
No. of restraints	3
H-atom treatment	H atoms treated by a mixture of independent and constrained refinement
Δρ_max_, Δρ_min_ (e Å^−3^)	2.21, −1.04

Computer programs: *PAL BL2D-SMDC* (Shin *et al.*, 2016 ▸), *HKL3000sm* (Otwinowski & Minor, 1997 ▸), *SHELXT2015* (Sheldrick, 2015*a* ▸), *SHELXL2015* (Sheldrick, 2015*b* ▸), *DIAMOND* (Putz & Brandenburg, 2014 ▸) and *publCIF* (Westrip, 2010 ▸).

## supplementary crystallographic information

### Crystal data

**Table:**

(C_10_H_28_N_4_)[Cr_2_O_7_]_2_·H_2_O	*F*(000) = 1336
*M**_r_* = 654.38	*D*_x_ = 1.934 Mg m^−^^3^
Monoclinic, *P*2_1_/*c*	Synchrotron radiation, λ = 0.610 Å
*a* = 10.428 (2) Å	Cell parameters from 70448 reflections
*b* = 13.961 (2) Å	θ = 0.4–33.7°
*c* = 15.490 (2) Å	µ = 1.28 mm^−^^1^
β = 94.671 (3)°	*T* = 200 K
*V* = 2247.6 (6) Å^3^	Block, yellow
*Z* = 4	0.11 × 0.10 × 0.09 mm

### Data collection

**Table:**

ADSC Q210 CCD area detector diffractometer	5804 reflections with *I* > 2σ(*I*)
Radiation source: PLSII 2D bending magnet	*R*_int_ = 0.016
ω scan	θ_max_ = 25.5°, θ_min_ = 1.7°
Absorption correction: empirical (using intensity measurements) (*HKL3000sm Scalepack*; Otwinowski & Minor, 1997)	*h* = −14→14
*T*_min_ = 0.942, *T*_max_ = 1.000	*k* = −19→19
12953 measured reflections	*l* = −21→21
6614 independent reflections	

### Refinement

**Table:**

Refinement on *F*^2^	Hydrogen site location: mixed
Least-squares matrix: full	H atoms treated by a mixture of independent and constrained refinement
*R*[*F*^2^ > 2σ(*F*^2^)] = 0.038	*w* = 1/[σ^2^(*F*_o_^2^) + (0.0712*P*)^2^ + 4.2149*P*] where *P* = (*F*_o_^2^ + 2*F*_c_^2^)/3
*wR*(*F*^2^) = 0.124	(Δ/σ)_max_ = 0.001
*S* = 1.09	Δρ_max_ = 2.21 e Å^−^^3^
6614 reflections	Δρ_min_ = −1.04 e Å^−^^3^
307 parameters	Extinction correction: SHELXL2016 (Sheldrick, 2015*b*), Fc^*^=kFc[1+0.001xFc^2^λ^3^/sin(2θ)]^-1/4^
3 restraints	Extinction coefficient: 0.0096 (10)

### Special details

**Table:**

Geometry. All esds (except the esd in the dihedral angle between two l.s. planes) are estimated using the full covariance matrix. The cell esds are taken into account individually in the estimation of esds in distances, angles and torsion angles; correlations between esds in cell parameters are only used when they are defined by crystal symmetry. An approximate (isotropic) treatment of cell esds is used for estimating esds involving l.s. planes.

### Fractional atomic coordinates and isotropic or equivalent isotropic displacement parameters (Å^2^)

**Table:**

	*x*	*y*	*z*	*U*_iso_*/*U*_eq_	
Cr1A	0.94583 (4)	0.25171 (3)	0.36481 (2)	0.00893 (10)	
Cr2A	0.97052 (4)	0.12932 (3)	0.18519 (2)	0.01054 (10)	
O1A	0.94853 (19)	0.23337 (12)	0.25072 (11)	0.0152 (3)	
O2A	0.9464 (2)	0.16272 (14)	0.08541 (12)	0.0215 (4)	
O3A	0.8720 (2)	0.04430 (14)	0.20636 (13)	0.0198 (4)	
O4A	1.1160 (2)	0.09255 (16)	0.20477 (14)	0.0239 (4)	
O5A	1.05842 (18)	0.18684 (14)	0.41408 (13)	0.0198 (4)	
O6A	0.80867 (18)	0.22499 (15)	0.39940 (13)	0.0207 (4)	
O7A	0.9714 (2)	0.36520 (13)	0.37925 (12)	0.0189 (4)	
Cr1B	0.49741 (4)	0.58036 (3)	0.18882 (2)	0.01299 (10)	
Cr2B	0.45749 (4)	0.72591 (3)	0.35345 (2)	0.01274 (10)	
O1B	0.5392 (2)	0.66545 (16)	0.27369 (13)	0.0226 (4)	
O2B	0.3859 (2)	0.51137 (17)	0.21811 (15)	0.0283 (5)	
O3B	0.6245 (2)	0.51689 (16)	0.17659 (13)	0.0244 (4)	
O4B	0.4512 (2)	0.63565 (15)	0.09915 (13)	0.0225 (4)	
O5B	0.5562 (3)	0.8034 (2)	0.39672 (19)	0.0485 (8)	
O6B	0.4180 (2)	0.65172 (15)	0.42912 (13)	0.0222 (4)	
O7B	0.3338 (3)	0.7803 (2)	0.31136 (17)	0.0442 (7)	
N1C	1.1608 (2)	0.48518 (17)	0.07746 (16)	0.0207 (4)	
H1NC	1.192696	0.527486	0.118455	0.025*	
H2NC	1.084404	0.508441	0.053719	0.025*	
N2C	0.8491 (2)	0.38005 (16)	0.07391 (15)	0.0179 (4)	
H3NC	0.905802	0.373982	0.032598	0.021*	
H4NC	0.829751	0.320363	0.092561	0.021*	
C1C	1.2545 (2)	0.47649 (17)	0.00725 (15)	0.0133 (4)	
H1C1	1.338852	0.453735	0.033274	0.016*	
H1C2	1.221515	0.428903	−0.036475	0.016*	
C2C	1.1387 (2)	0.38925 (17)	0.11984 (15)	0.0129 (4)	
H2C1	1.110865	0.341470	0.074936	0.016*	
H2C2	1.219961	0.366488	0.150578	0.016*	
C3C	1.0374 (2)	0.39918 (15)	0.18280 (12)	0.0071 (3)	
H3C1	1.022978	0.335164	0.207745	0.008*	
H3C2	1.072279	0.440867	0.230818	0.008*	
C4C	0.9100 (2)	0.43839 (17)	0.14937 (15)	0.0125 (4)	
H4C1	0.920930	0.505402	0.130394	0.015*	
H4C2	0.851635	0.438789	0.196698	0.015*	
C5C	0.72907 (19)	0.42821 (15)	0.03596 (13)	0.0068 (3)	
H5C1	0.671037	0.437307	0.082792	0.008*	
H5C2	0.685296	0.384273	−0.007113	0.008*	
N1D	0.74042 (19)	0.43291 (15)	0.45783 (13)	0.0124 (4)	
H1ND	0.797863	0.387321	0.444322	0.015*	
H2ND	0.694753	0.408892	0.500537	0.015*	
N2D	0.6336 (2)	0.57400 (17)	0.58080 (16)	0.0213 (5)	
H3ND	0.589300	0.521117	0.561168	0.026*	
H4ND	0.676852	0.559214	0.632478	0.026*	
C1D	0.5652 (2)	0.36748 (17)	0.35112 (15)	0.0139 (4)	
H1D1	0.527317	0.380206	0.291539	0.017*	
H1D2	0.619964	0.309743	0.348804	0.017*	
C2D	0.6505 (2)	0.45172 (18)	0.38007 (15)	0.0131 (4)	
H2D1	0.701386	0.470585	0.331598	0.016*	
H2D2	0.594811	0.506624	0.392587	0.016*	
C3D	0.8139 (2)	0.52031 (18)	0.49225 (16)	0.0144 (4)	
H3D1	0.870329	0.542564	0.447912	0.017*	
H3D2	0.869859	0.501556	0.544205	0.017*	
C4D	0.7290 (2)	0.60301 (18)	0.51601 (16)	0.0142 (4)	
H4D1	0.680965	0.627588	0.462816	0.017*	
H4D2	0.783948	0.655524	0.541016	0.017*	
C5D	0.5418 (2)	0.65411 (15)	0.59291 (14)	0.0084 (4)	
H5D1	0.503368	0.674117	0.535164	0.010*	
H5D2	0.591171	0.709254	0.618333	0.010*	
O1W	0.7376 (2)	0.75592 (18)	0.19670 (18)	0.0313 (5)	
H1O1	0.749 (13)	0.723 (5)	0.152 (4)	0.30 (8)*	
H2O1	0.708 (5)	0.718 (3)	0.232 (3)	0.066 (17)*	

### Atomic displacement parameters (Å^2^)

**Table:**

	*U*^11^	*U*^22^	*U*^33^	*U*^12^	*U*^13^	*U*^23^
Cr1A	0.01239 (17)	0.00745 (17)	0.00736 (17)	0.00232 (12)	0.00323 (12)	0.00134 (12)
Cr2A	0.01754 (19)	0.00764 (17)	0.00646 (17)	0.00091 (13)	0.00113 (12)	−0.00038 (12)
O1A	0.0271 (9)	0.0096 (7)	0.0094 (7)	0.0002 (7)	0.0048 (6)	−0.0016 (6)
O2A	0.0375 (11)	0.0171 (9)	0.0098 (8)	0.0022 (8)	0.0012 (7)	0.0016 (7)
O3A	0.0267 (10)	0.0128 (8)	0.0197 (9)	−0.0043 (7)	−0.0002 (7)	0.0030 (7)
O4A	0.0219 (9)	0.0260 (10)	0.0238 (10)	0.0072 (8)	0.0012 (8)	−0.0015 (8)
O5A	0.0186 (9)	0.0214 (9)	0.0194 (9)	0.0085 (7)	0.0013 (7)	0.0053 (7)
O6A	0.0161 (8)	0.0260 (10)	0.0209 (9)	0.0013 (7)	0.0059 (7)	0.0078 (8)
O7A	0.0306 (10)	0.0104 (8)	0.0159 (8)	0.0000 (7)	0.0036 (7)	−0.0028 (6)
Cr1B	0.01447 (19)	0.01425 (19)	0.01028 (18)	0.00271 (13)	0.00115 (13)	−0.00104 (13)
Cr2B	0.0189 (2)	0.01113 (18)	0.00895 (17)	0.00155 (13)	0.00592 (13)	0.00160 (13)
O1B	0.0241 (9)	0.0264 (10)	0.0181 (9)	−0.0004 (8)	0.0067 (7)	−0.0098 (8)
O2B	0.0254 (10)	0.0306 (11)	0.0292 (11)	−0.0093 (9)	0.0038 (8)	0.0042 (9)
O3B	0.0250 (10)	0.0290 (11)	0.0194 (9)	0.0134 (8)	0.0023 (7)	−0.0046 (8)
O4B	0.0276 (10)	0.0218 (10)	0.0171 (9)	0.0010 (8)	−0.0051 (7)	0.0036 (7)
O5B	0.0656 (19)	0.0379 (14)	0.0446 (15)	−0.0317 (14)	0.0206 (14)	−0.0224 (12)
O6B	0.0291 (10)	0.0231 (10)	0.0149 (8)	−0.0037 (8)	0.0040 (7)	0.0079 (7)
O7B	0.0396 (14)	0.0612 (18)	0.0347 (13)	0.0314 (13)	0.0199 (11)	0.0275 (13)
N1C	0.0239 (11)	0.0177 (10)	0.0207 (10)	0.0008 (9)	0.0035 (8)	0.0042 (9)
N2C	0.0230 (11)	0.0143 (10)	0.0165 (10)	−0.0009 (8)	0.0028 (8)	−0.0007 (8)
C1C	0.0150 (10)	0.0110 (10)	0.0140 (10)	0.0001 (8)	0.0020 (8)	0.0013 (8)
C2C	0.0151 (10)	0.0113 (10)	0.0125 (9)	0.0006 (8)	0.0011 (8)	0.0028 (8)
C3C	0.0131 (9)	0.0052 (8)	0.0028 (8)	−0.0017 (7)	0.0005 (7)	0.0022 (6)
C4C	0.0161 (10)	0.0119 (10)	0.0098 (9)	0.0001 (8)	0.0019 (8)	−0.0017 (8)
C5C	0.0083 (8)	0.0076 (8)	0.0046 (8)	−0.0029 (7)	0.0020 (6)	0.0005 (7)
N1D	0.0140 (9)	0.0138 (9)	0.0096 (8)	0.0049 (7)	0.0021 (7)	0.0015 (7)
N2D	0.0226 (11)	0.0189 (11)	0.0228 (11)	0.0038 (9)	0.0039 (9)	0.0000 (9)
C1D	0.0164 (10)	0.0149 (10)	0.0106 (10)	0.0037 (8)	0.0017 (8)	−0.0030 (8)
C2D	0.0136 (10)	0.0159 (10)	0.0098 (9)	0.0021 (8)	0.0000 (7)	0.0024 (8)
C3D	0.0108 (9)	0.0183 (11)	0.0141 (10)	0.0000 (8)	0.0021 (8)	0.0024 (9)
C4D	0.0148 (10)	0.0128 (10)	0.0152 (10)	−0.0017 (8)	0.0019 (8)	−0.0002 (8)
C5D	0.0107 (9)	0.0054 (8)	0.0093 (9)	0.0017 (7)	0.0018 (7)	−0.0019 (7)
O1W	0.0229 (10)	0.0311 (12)	0.0405 (13)	0.0049 (9)	0.0053 (9)	0.0165 (10)

### Geometric parameters (Å, º)

**Table:**

Cr1A—O6A	1.6114 (19)	C3C—C4C	1.491 (3)
Cr1A—O7A	1.6192 (19)	C3C—H3C1	0.9900
Cr1A—O5A	1.6233 (19)	C3C—H3C2	0.9900
Cr1A—O1A	1.7882 (18)	C4C—H4C1	0.9900
Cr2A—O4A	1.607 (2)	C4C—H4C2	0.9900
Cr2A—O2A	1.6143 (19)	C5C—H5C1	0.9900
Cr2A—O3A	1.6209 (19)	C5C—H5C2	0.9900
Cr2A—O1A	1.7975 (18)	N1D—C2D	1.489 (3)
Cr1B—O2B	1.603 (2)	N1D—C3D	1.515 (3)
Cr1B—O3B	1.618 (2)	N1D—H1ND	0.9100
Cr1B—O4B	1.627 (2)	N1D—H2ND	0.9100
Cr1B—O1B	1.799 (2)	N2D—C5D	1.494 (3)
Cr2B—O7B	1.590 (2)	N2D—C4D	1.524 (3)
Cr2B—O5B	1.602 (3)	N2D—H3ND	0.9100
Cr2B—O6B	1.6417 (19)	N2D—H4ND	0.9100
Cr2B—O1B	1.7711 (19)	C1D—C5D^ii^	1.498 (3)
N1C—C2C	1.517 (3)	C1D—C2D	1.520 (3)
N1C—C1C	1.524 (3)	C1D—H1D1	0.9900
N1C—H1NC	0.9100	C1D—H1D2	0.9900
N1C—H2NC	0.9100	C2D—H2D1	0.9900
N2C—C5C	1.498 (3)	C2D—H2D2	0.9900
N2C—C4C	1.521 (3)	C3D—C4D	1.519 (3)
N2C—H3NC	0.9100	C3D—H3D1	0.9900
N2C—H4NC	0.9100	C3D—H3D2	0.9900
C1C—C5C^i^	1.506 (3)	C4D—H4D1	0.9900
C1C—H1C1	0.9900	C4D—H4D2	0.9900
C1C—H1C2	0.9900	C5D—H5D1	0.9900
C2C—C3C	1.501 (3)	C5D—H5D2	0.9900
C2C—H2C1	0.9900	O1W—H1O1	0.844 (10)
C2C—H2C2	0.9900	O1W—H2O1	0.844 (10)
			
O6A—Cr1A—O7A	108.74 (11)	C3C—C4C—N2C	112.02 (19)
O6A—Cr1A—O5A	110.00 (10)	C3C—C4C—H4C1	109.2
O7A—Cr1A—O5A	112.10 (11)	N2C—C4C—H4C1	109.2
O6A—Cr1A—O1A	112.41 (10)	C3C—C4C—H4C2	109.2
O7A—Cr1A—O1A	105.13 (9)	N2C—C4C—H4C2	109.2
O5A—Cr1A—O1A	108.41 (9)	H4C1—C4C—H4C2	107.9
O4A—Cr2A—O2A	110.11 (11)	N2C—C5C—C1C^i^	116.69 (18)
O4A—Cr2A—O3A	109.41 (11)	N2C—C5C—H5C1	108.1
O2A—Cr2A—O3A	110.70 (11)	C1C^i^—C5C—H5C1	108.1
O4A—Cr2A—O1A	108.27 (10)	N2C—C5C—H5C2	108.1
O2A—Cr2A—O1A	106.87 (9)	C1C^i^—C5C—H5C2	108.1
O3A—Cr2A—O1A	111.43 (10)	H5C1—C5C—H5C2	107.3
Cr1A—O1A—Cr2A	133.37 (11)	C2D—N1D—C3D	114.21 (19)
O2B—Cr1B—O3B	108.89 (13)	C2D—N1D—H1ND	108.7
O2B—Cr1B—O4B	110.81 (11)	C3D—N1D—H1ND	108.7
O3B—Cr1B—O4B	110.38 (11)	C2D—N1D—H2ND	108.7
O2B—Cr1B—O1B	109.15 (11)	C3D—N1D—H2ND	108.7
O3B—Cr1B—O1B	107.16 (10)	H1ND—N1D—H2ND	107.6
O4B—Cr1B—O1B	110.35 (11)	C5D—N2D—C4D	110.1 (2)
O7B—Cr2B—O5B	108.74 (19)	C5D—N2D—H3ND	109.6
O7B—Cr2B—O6B	110.59 (12)	C4D—N2D—H3ND	109.6
O5B—Cr2B—O6B	108.50 (14)	C5D—N2D—H4ND	109.6
O7B—Cr2B—O1B	111.22 (12)	C4D—N2D—H4ND	109.6
O5B—Cr2B—O1B	106.51 (13)	H3ND—N2D—H4ND	108.2
O6B—Cr2B—O1B	111.12 (11)	C5D^ii^—C1D—C2D	115.47 (19)
Cr2B—O1B—Cr1B	136.28 (12)	C5D^ii^—C1D—H1D1	108.4
C2C—N1C—C1C	111.5 (2)	C2D—C1D—H1D1	108.4
C2C—N1C—H1NC	109.3	C5D^ii^—C1D—H1D2	108.4
C1C—N1C—H1NC	109.3	C2D—C1D—H1D2	108.4
C2C—N1C—H2NC	109.3	H1D1—C1D—H1D2	107.5
C1C—N1C—H2NC	109.3	N1D—C2D—C1D	114.7 (2)
H1NC—N1C—H2NC	108.0	N1D—C2D—H2D1	108.6
C5C—N2C—C4C	110.15 (18)	C1D—C2D—H2D1	108.6
C5C—N2C—H3NC	109.6	N1D—C2D—H2D2	108.6
C4C—N2C—H3NC	109.6	C1D—C2D—H2D2	108.6
C5C—N2C—H4NC	109.6	H2D1—C2D—H2D2	107.6
C4C—N2C—H4NC	109.6	N1D—C3D—C4D	114.17 (19)
H3NC—N2C—H4NC	108.1	N1D—C3D—H3D1	108.7
C5C^i^—C1C—N1C	110.47 (19)	C4D—C3D—H3D1	108.7
C5C^i^—C1C—H1C1	109.6	N1D—C3D—H3D2	108.7
N1C—C1C—H1C1	109.6	C4D—C3D—H3D2	108.7
C5C^i^—C1C—H1C2	109.6	H3D1—C3D—H3D2	107.6
N1C—C1C—H1C2	109.6	C3D—C4D—N2D	112.5 (2)
H1C1—C1C—H1C2	108.1	C3D—C4D—H4D1	109.1
C3C—C2C—N1C	109.84 (19)	N2D—C4D—H4D1	109.1
C3C—C2C—H2C1	109.7	C3D—C4D—H4D2	109.1
N1C—C2C—H2C1	109.7	N2D—C4D—H4D2	109.1
C3C—C2C—H2C2	109.7	H4D1—C4D—H4D2	107.8
N1C—C2C—H2C2	109.7	N2D—C5D—C1D^ii^	115.92 (19)
H2C1—C2C—H2C2	108.2	N2D—C5D—H5D1	108.3
C4C—C3C—C2C	117.56 (18)	C1D^ii^—C5D—H5D1	108.3
C4C—C3C—H3C1	107.9	N2D—C5D—H5D2	108.3
C2C—C3C—H3C1	107.9	C1D^ii^—C5D—H5D2	108.3
C4C—C3C—H3C2	107.9	H5D1—C5D—H5D2	107.4
C2C—C3C—H3C2	107.9	H1O1—O1W—H2O1	106 (3)
H3C1—C3C—H3C2	107.2		
			
O6A—Cr1A—O1A—Cr2A	−81.13 (17)	C2C—N1C—C1C—C5C^i^	177.91 (19)
O7A—Cr1A—O1A—Cr2A	160.74 (15)	C1C—N1C—C2C—C3C	−175.00 (19)
O5A—Cr1A—O1A—Cr2A	40.70 (18)	N1C—C2C—C3C—C4C	55.8 (3)
O4A—Cr2A—O1A—Cr1A	−67.38 (18)	C2C—C3C—C4C—N2C	56.8 (3)
O2A—Cr2A—O1A—Cr1A	174.04 (15)	C5C—N2C—C4C—C3C	−173.51 (18)
O3A—Cr2A—O1A—Cr1A	52.99 (18)	C4C—N2C—C5C—C1C^i^	65.9 (2)
O7B—Cr2B—O1B—Cr1B	−53.2 (2)	C3D—N1D—C2D—C1D	−173.36 (19)
O5B—Cr2B—O1B—Cr1B	−171.5 (2)	C5D^ii^—C1D—C2D—N1D	73.5 (3)
O6B—Cr2B—O1B—Cr1B	70.5 (2)	C2D—N1D—C3D—C4D	57.5 (3)
O2B—Cr1B—O1B—Cr2B	−32.7 (2)	N1D—C3D—C4D—N2D	55.5 (3)
O3B—Cr1B—O1B—Cr2B	−150.48 (18)	C5D—N2D—C4D—C3D	−172.66 (19)
O4B—Cr1B—O1B—Cr2B	89.3 (2)	C4D—N2D—C5D—C1D^ii^	174.65 (19)

Symmetry codes: (i) −*x*+2, −*y*+1, −*z*; (ii) −*x*+1, −*y*+1, −*z*+1.

### Hydrogen-bond geometry (Å, º)

**Table:**

*D*—H···*A*	*D*—H	H···*A*	*D*···*A*	*D*—H···*A*
N1*C*—H1*NC*···O2*B*^iii^	0.91	2.45	3.091 (3)	128
N1*D*—H1*ND*···O6*A*	0.91	2.38	3.140 (3)	142
N1*D*—H1*ND*···O7*A*	0.91	2.16	2.942 (3)	143
N1*D*—H2*ND*···O6*B*^ii^	0.91	1.87	2.768 (3)	169
N2*C*—H3*NC*···O6*A*^iv^	0.91	2.62	3.074 (3)	112
N2*C*—H4*NC*···O7*B*^v^	0.91	2.42	3.047 (3)	126
N2*C*—H4*NC*···O2*A*	0.91	2.52	3.200 (3)	132
N2*D*—H3*ND*···O6*B*^ii^	0.91	2.42	3.198 (3)	144
N2*D*—H4*ND*···O2*B*^ii^	0.91	2.65	3.357 (3)	136
O1*W*—H1*O*1···O5*A*^vi^	0.84 (1)	2.38 (10)	2.999 (3)	130 (11)
O1*W*—H2*O*1···O1*B*	0.84 (1)	2.05 (4)	2.774 (3)	143 (5)

Symmetry codes: (ii) −*x*+1, −*y*+1, −*z*+1; (iii) *x*+1, *y*, *z*; (iv) *x*, −*y*+1/2, *z*−1/2; (v) −*x*+1, *y*−1/2, −*z*+1/2; (vi) −*x*+2, *y*+1/2, −*z*+1/2.
